# *EWS-Oct-4B*, an alternative *EWS-Oct-4* fusion gene, is a potent oncogene linked to human epithelial tumours

**DOI:** 10.1038/sj.bjc.6605516

**Published:** 2010-01-05

**Authors:** S Kim, B Lim, J Kim

**Affiliations:** 1 Laboratory of Molecular and Cellular Biology, Department of Life Science, Sogang University, Seoul 121-742, Korea

**Keywords:** EWS-Oct-4B, alternative fusion gene, chromosomal translocation, chimeric protein, transformation, epithelial tumours

## Abstract

**Background:**

Characterisation of EWS-Oct-4 translocation fusion product in bone and soft-tissue tumours revealed a chimeric gene resulting from an in-frame fusion between *EWS (Ewing's sarcoma gene)* exons 1–6 and *Oct-4* exons 1–4. Recently, an alternative form of the fusion protein between the *EWS* and *Oct-4* genes, named EWS-Oct-4B, was reported in two types of epithelial tumours, a hidradenoma of the skin and a mucoepidermoid carcinoma of the salivary glands. As the N-terminal and POU domains of the EWS-Oct-4 and EWS-Oct-4B proteins are not structurally identical, we decided to investigate the functional consequences of the EWS-Oct-4B fusion.

**Methods:**

In this report, we have characterised the EWS-Oct-4B fusion protein. To investigate how the EWS-Oct-4B protein contributes to tumourigenesis in human cancers, we analysed its DNA-binding activity, subcellular localisation, transcriptional activation behaviour, and oncogenic properties.

**Results:**

We found that this new chimeric gene encodes a nuclear protein that binds DNA with the same sequence specificity as the parental Oct-4 protein or the fusion EWS-Oct-4 protein. We show that the nuclear localisation signal of EWS-Oct-4B is dependent on the POU DNA-binding domain, and we identified a cluster of basic amino acids, ^269^RKRKR^273^, in the POU domain that specifically mediates the nuclear localisation of EWS-Oct-4B. Comparison of the properties of EWS-Oct-4B and EWS-Oct-4 indicated that EWS-Oct-4B is a less-potent transcriptional activator of a reporter construct carrying the Oct-4-binding sites. Deletion analysis of the functional domains of EWS-Oct-4B revealed that the EWS N-terminal domain (NTD)^B^, POU, and C-terminal domain (CTD) are necessary for its full transactivation potential. Despite its reduced activity as a transcriptional activator, EWS-Oct-4B regulated the expression of *fgf-4* (fibroblast growth factor-4) and *nanog*, which are potent mitogens, as well as of Oct-4 downstream target genes, the promoters of which contain potential Oct-4-binding sites. Finally, ectopic expression of *EWS-Oct-4B* in Oct-4-null ZHBTc4 ES cells resulted in increased tumourigenic growth potential in nude mice.

**Conclusion:**

These results suggest that the oncogenic effect of the t(6;22) translocation is due to the EWS-Oct-4B chimeric protein, and that alternative fusion of the EWS amino terminal domain to the Oct-4 DNA-binding domain produces another transforming chimeric product in human epithelial tumours.

Cancer is an abnormal type of tissue growth in which cells proliferate in an uncontrolled, relatively autonomous manner, leading to a continual increase in the number of dividing cells. Chromosomal translocations cause cancer by activating existing genes or creating new fusion proteins (Rabbitts, 1999). Some bone and soft-tissue tumours were shown to harbour a translocation t(6;22)(p21;q12) involving Ewing's sarcoma (*EWS*) gene at 22q12 and the *Oct-4* gene at 6p21 (Yamaguchi *et al*, 2005). Striking features of these tumours are the diffuse proliferation pattern of the undifferentiated tumour cells and the positive immunoreactivity for vimentin, S-100, and neuron-specific enolase. A chimeric transcript of ∼1.8 kb was detected by northern blotting using *EWS* and *Oct-4* probes, and an *EWS-Oct-4* fusion transcript, but not the reciprocal *Oct-4-EWS* fusion transcript, was detected in tumours by RT–PCR (Yamaguchi *et al*, 2005). Recently, a new fusion between *EWS* and *Oct-4* was reported in hidradenoma of the skin and mucoepidermoid carcinoma of the salivary glands (Moller *et al*, 2008). Reverse transcription-PCR and DNA sequence analyses revealed that a part of exon 6 of *EWS* is fused in-frame to exon 2 of *Oct-4* in these tumours, which indicated that an alternative form of *EWS-Oct-4* exists owing to a variation in the locations of the *EWS* and *Oct-4* genomic break points. To distinguish it from the previous EWS-Oct-4 fusion detected in human bone and soft-tissue tumours (Yamaguchi *et al*, 2005), we called it EWS-Oct-4B.

The *EWS* gene encodes a 656 amino acid protein that contains three arginine- and glycine-rich tracts and an 85 amino acid RNA recognition motif at its C-terminus. The N-terminal domain (NTD) (amino acid 1–285) of EWS is composed almost exclusively (∼90%) of tyrosine, glycine, alanine, serine, threonine, and proline residues organised in a repeated and degenerate polypeptide motif with the consensus, NSYGQQS. This domain has weak homology to the C-terminal region of eukaryotic RNA polymerase II (Delattre *et al*, 1992). The *EWS* gene is involved in several tumour-related translocations, which generate fusions with genes for putative transcription factors (Kim and Pelletier, 1999). In each case, the translocation produces chimeric molecules containing the NTD of EWS fused to the DNA-binding domain of the partner protein. The C-terminal fusion partners are cellular transcription factors that contribute a sequence-specific DNA-binding domain, which determines the tumour phenotype (Lee, 2007). The highly tissue-restricted expression of the fusion partners contrasts with that of the native *EWS* gene, which is expressed ubiquitously at high levels. The *EWS* promoter drives the expression of EWS fusion proteins directly in human cancers owing to the genomic structure of the EWS chimeras (Lin *et al*, 1999).

Oct-4, also referred to as Oct-3, is a transcriptional regulator of genes involved in maintaining the undifferentiated pluripotent state, and may prevent the expression of genes activated during differentiation (Brehm *et al*, 1997). It functions as a master switch during differentiation by regulating gene expression in pluripotent cells, or in cells that can develop pluripotent potential (Ovitt and Scholer, 1998; Pesce and Scholer, 2001). In addition, Oct-4 is a key factor in the genesis of human testicular germ cell tumours (TGCTs) (Gidekel *et al*, 2003; Looijenga *et al*, 2003). Human TGCTs are the most common malignancy in adolescent and young adult Caucasian males, and are the cause of one in seven deaths in this group (Oliver, 1999; Hellerstedt and Pienta, 2003). The Oct-4 transcript is consistently detected in a specific set of human TGCTs found in adolescents and young adults, namely, seminomas and embryonal carcinomas (Palumbo *et al*, 2002). In addition, the precursor lesions of human TGCT, known as carcinoma *in situ*, also express *Oct-4* (Palumbo *et al*, 2002). Expression of *Oct-4* has also been reported in human primary breast carcinomas, human breast cancer cell lines, and other types of carcinoma cell lines, suggesting that its involvement in tumourigenesis may be related to the upregulation of its downstream target genes (Jin *et al*, 1999; Monk and Holding, 2001; Gidekel *et al*, 2003; Wang *et al*, 2003). Consistent with these findings, *Oct-4* expression in a heterologous cell system transformed nontumourigenic cells and produced tumours in nude mice. Activation of *Oct-4* in adult mice using a doxycycline-dependent expression system resulted in dysplastic growth of epithelial tissues that are dependent on continuous *Oct-4* expression (Hochedlinger *et al*, 2005). In addition, Oct-4 is involved in the reprogramming of mouse and human somatic cells to the pluripotent state (Takahashi and Yamanaka, 2006; Okita *et al*, 2007; Takahashi *et al*, 2007; Wernig *et al*, 2007, 2008; Aasen *et al*, 2008; Dimos *et al*, 2008; Hockemeyer *et al*, 2008; Lowry *et al*, 2008; Maherali *et al*, 2008; Nakagawa *et al*, 2008; Park *et al*, 2008a, 2008b), suggesting that induced pluripotent stem cells, derived from somatic cells of patients, represent a powerful tool for use in replacement therapies (Soldner *et al*, 2009).

To investigate how the recently identified EWS-Oct-4B protein contributes to tumourigenesis in two types of human epithelial tumours, namely, hidradenoma of the skin and mucoepidermoid carcinoma of the salivary glands, we analysed its transcriptional activation behaviour and oncogenic properties. We found that it is a nuclear protein that binds DNA with a sequence specificity indistinguishable from that of the parental Oct-4 or the chimeric EWS-Oct-4 proteins. The nuclear localisation signal (NLS) of EWS-Oct-4B was dependent on the POU DNA-binding domain, and we identified a cluster of basic amino acids, ^269^RKRKR^273^, in the POU domain that specifically mediates the nuclear localisation of EWS-Oct-4B. When we compared transactivation of reporter constructs carrying Oct-4-binding sites by the different EWS-Oct-4 proteins, EWS-Oct-4B was a less-potent transcriptional activator than EWS-Oct-4. Results from deletion analysis also suggested that several functional domains of EWS-Oct-4B are necessary for it to achieve its full activation potential. However, although EWS-Oct-4B is a less-potent transcriptional activator than EWS-Oct-4, it functions as a dominantly acting oncogene, as measured by the activation of oncogenic Oct-4 downstream target genes and tumour formation in nude mice. These data indicate that EWS-Oct-4B may have a critical role in the formation of human epithelial tumours by activating the transcription of Oct-4 target genes.

## Materials and methods

### Molecular cloning

To generate pcDNA3/Flag-EWS-Oct-4B, we used the QuikChange site-directed mutagenesis kit (Stratagene, La Jolla, CA, USA), the mutagenic primer set 5′-EOB (5′-AACTACAGTTATCCCCAGTCCCAGGACATCAAAGCTC-3′) and 3′-EOB (5′-ACGTTTGATGTCCTGGGACTGGGGATAACTGTAGTTAC-3′), and pKSII/Flag-EWS-Oct-4 (Lee *et al*, 2007) as a template to construct pKSII/Flag-EWS-Oct-4B. Then, pKSII/Flag-EWS-Oct-4B was digested with *Eco*RI and *Hin*dIII and cloned into the corresponding sites of the pcDNA3 vector (Invitrogen Molecular Probes, Carlsbad, USA).

The GST-EGFP-EWS-Oct-4B deletion mutants were generated as follows: (A) pGST-EGFP, details on the construction of pGST-EGFP have been previously reported (Kim *et al*, 2008); (B) pGST-EGFP/EWS (NTD)^B^, an EWS (NTD)^B^ fragment was amplified from pcDNA3/EWS-Oct-4B by PCR using primers 5′-GST-EGFP-EWS(N) (5′-GATCGGATCCAGCGTCCACGGATTACAG-3′ *Bam*HI site underlined) and 3′-GST-EGFP-EWS(N) (5′-GATCGGATCCAGCTGGGGATAACTGTAG-3′ *Bam*HI site underlined), digested with *Bam*HI, and cloned into the corresponding site of the pGST-EGFP vector to generate pGST-EGFP/EWS (NTD)^B^; (C) pGST-EGFP/POU^B^, a POU^B^ fragment was amplified from pcDNA3/EWS-Oct-4B by PCR using primers 5′-GST-EGFP-POU (5′-GATCGGATCCATCCCAGGACATCAAAGC-3′ *Bam*HI site underlined) and 3′-GST-EGFP-POU (5′-GATCGGATCCAGGCTTGATCGCTTGCCC-3′ *Bam*HI site underlined), digested with *Bam*HI, and cloned into the corresponding site of the pGST-EGFP vector to generate pGST-EGFP/POU^B^; (D) pGST-EGFP-POU^B^ (LILIL), pcDNA3/EWS-Oct-4B (LILIL), in which amino acids ^269^RKRKR^273^ were substituted with ^269^LILIL^273^, was generated using the QuikChange site-directed mutagenesis kit (Stratagene) and the mutagenic primer set 5′-mNLS (5′-CCCTCGTGCAGGCCCTAATCCTA ATCCTAACCAGTATCGAGAAC-3′) and 3′-mNLS (5′-GTTCTCGATACTGGTTAGGATTA GGATTAGGGCCTGCACGAGGG-3′). To construct pGST-EGFP/POU^B^ (LILIL), POU^B^ (LILIL) was amplified from pcDNA3/EWS-Oct-4B (LILIL) by PCR using primers 5′-GST-EGFP-POU and 3′-GST-EGFP-POU, digested with *Bam*HI, and cloned into the corresponding site of pGST-EGFP; (E) pGST-EGFP/CTD, a C-terminal domain (CTD) fragment was amplified from pcDNA3/EWS-Oct-4B by PCR using primers 5′-GST-EGFP-CTD (5′-GATCGGATCCAAGCGACTATGCACAACG-3′ *Bam*HI site underlined) and 3′-GST-EGFP-CTD (5′-GATCGGATCCAGGTTTGAATGCATGGGA-3′ *Bam*HI site underlined), digested with *Bam*HI, and cloned into the corresponding site of the pGST-EGFP vector to generate pGST-EGFP/CTD.

To construct pGAL4-EWS (NTD), an EWS fragment (amino acid 1–193) was amplified from pcDNA3/Flag-EWS-Oct-4 by PCR using primers 5′-E-OFlagEcoRI (5′-GATCGAATTCATGGATTACAAGGATGAC-3′ *Eco*RI site underlined) and 3′-EWSBamHI (5′-GATCGGATCCTAGGTAGGAGGGTAGGATGG-3′ *Bam*HI site underlined), digested with *Eco*RI and *Bam*HI, and cloned into the corresponding sites of the pM vector (Clontech Laboratories, Mountain View, CA, USA). To construct pGAL4-EWS (NTD)^B^, an EWS fragment (amino acid 1–174) was amplified from pcDNA3/Flag-EWS-Oct-4 by PCR using primers 5′-E-OFlagEcoRI and 3′-EWS(B)BamHI (5′-GATCGGATCCCTGGGGATAACTGTAGTT-3′ *Bam*HI site underlined), digested with *Eco*RI and *Bam*HI, and cloned into the corresponding sites of the pM vector.

The pcDNA3/Flag-EWS-Oct-4B deletion mutants were generated as follows: (A) pcDNA3/Flag-EWS-Oct-4B (ΔEWS), a Flag-EWS-Oct-4B (ΔEWS) fragment was amplified from pKSII/Flag-EWS-Oct-4B by PCR using primers 5′-FlagOct-4BPOU (5′-GATCAAGCTTATGGATTACAAGGATGACGACGATAAGTC CCAGGACATCAAAGCTC-3′ *Hin*dIII site underlined) and 3′-EOEcoRI (5′-GATCGAATTCTCAGTTTGAATGCATGGG-3′ *Eco*RI site underlined), digested with *Hin*dIII and *Eco*RI, and cloned into the corresponding sites of the pcDNA3 vector to generate pcDNA3/Flag-EWS-Oct-4B (ΔEWS); (B) pcDNA3/Flag-EWS-Oct-4B (ΔCTD), a Flag-EWS-Oct-4B (ΔCTD) fragment was amplified from pKSII/Flag-EWS-Oct-4B by PCR using primers 5′-BamHIFlagE-O (5′-GATCGGATCCATGGATTACAAGGATGAC-3′ *Bam*HI site underlined) and 3′-hOct3A-867EcoRI (5′-GATCGAATTCGCTTGATCGCTTGCCCTT-3′ *Eco*RI site underlined), digested with *Bam*HI and *Eco*RI, and cloned into the corresponding sites of the pcDNA3 vector to generate pcDNA3/Flag-EWS-Oct-4B (ΔCTD); (C) pcDNA3/Flag-EWS-Oct-4B (V313P), pCAG-IP/EWS-Oct-4 (V351P) (Kim *et al*, 2009) was digested with *Eco*NI to isolate an EcoNI-V313P-EcoNI fragment. Then, pcDNA3/Flag-EWS-Oct-4B was digested with *Eco*NI to generate pcDNA3/Flag-EWS-Oct-4B (ΔEcoNI), and pcDNA3/Flag-EWS-Oct-4B (ΔEcoNI) was ligated with the EcoNI-V313P-EcoNI fragment to generate pcDNA3/Flag-EWS-Oct-4B (V313P).

Construct pCAG-IP/EGFP has been described previously (Lee *et al*, 2006). To create pCAG-IP/EWS-Oct-4B-EGFP, EWS-Oct-4B was amplified from pcDNA3/Flag-EWS-Oct-4B by PCR using primers 5′-BamHIFlagE-O (5′-GATCGGATCCATGGATTACAAGGATGAC-3′, *Bam*HI site underlined) and 3′-hOct4CTDGFP (5′-GATCGGATCCGCTCCGTTTGAATGCATGGG-3′, *Bam*HI site underlined). The PCR product was digested with *Bam*HI and cloned into the same sites of the pEGFP (N1) vector (Clontech Laboratories) to generate pEGFP(N1)-EWS-Oct-4B. To construct pCAG-IP/EWS-Oct-4B-EGFP, the blunted *Hin*dIII (repaired with the Klenow fragment) and *Not*I fragments of pEGFP(N1)-EWS-Oct-4B were subcloned into the blunted *Xho*I (repaired with the Klenow fragment) and *Not*I sites of the pCAG-IP vector.

### Electrophoretic mobility shift assays

Probes (5′-GGCACTTCACTAGCATAACAATGAGGGCTC-3′ and 5′-GAGCCCTCATTGTTATGCTAGTGAAGTGCC-3′ underlines indicate the Oct-4 recognition site) for electrophoretic mobility shift assays (EMSAs) were prepared from synthetic oligonucleotides for which the sequences have been described previously (Nishimoto *et al*, 1999). The probe was prepared by end-labelling annealed complementary oligonucleotides with [*γ*-^32^P] ATP using T4 polynucleotide kinase. The DNA-binding reactions were performed using purified glutathione-S-transferase (GST), GST-EWS-Oct-4, or GST-EWS-Oct-4B proteins for 30 min at 4°C in binding buffer containing 10 mM Tris-HCl (pH 8.0), 40 mM KCl, 6% glycerol, 1 mM DTT, 0.05% NP-40, and 10 ng *μ*l^−1^ of poly (dI dC) (dI dC). After binding, the reaction mixtures were run on 4% polyacrylamide gels (acrylamide/bisacrylamide ratio. 37 : 1) in 0.5 × TBE (44.5 mM Tris-HCl, 44.5 mM boric acid, 1 mM EDTA) buffer at 150 V for 2–3 h at 4°C. The gels were dried and exposed to Kodak X-Omat film (Kodak, Rochester, MN, USA) at −70°C, using an intensifying screen.

### Transfection, subcellular localisation, and reporter gene assays

Subcellular localisation experiments were performed as previously described (Kim *et al*, 2008). Briefly, to examine the localisation of the EWS-Oct-4B-EGFP or GST-EGFP fusion proteins of the EWS-Oct-4B truncation mutants, cells were transfected with pEGFP, pEWS-Oct-4B-EGFP, pGST-EGFP, pGST-EGFP/EWS (NTD)^B^, pGST-EGFP/POU^B^, pGST-EGFP/POU^B^ (LILIL), or pGST-EGFP/CTD using the VivaMagic reagent (Vivagen, Seoul, Korea), washed in PBS, and then fixed for 10 min at −20°C in a mixture of acetone and methanol (1 : 1, v/v). Coverslips were mounted with 50% glycerol/PBS, and the green fluorescence of EGFP was detected using a fluorescence microscope (IX71, Olympus, Tokyo, Japan) equipped with DP71 digital camera (Olympus). For reporter gene assays, cells were transiently transfected with plasmids by electroporation using the VivaMagic reagent (Vivagen). Luciferase assays were performed with the Dual-luciferase Assay System (Promega, Madison, WI, USA). *Renilla* luciferase activities were used to normalise transfection efficiencies.

### Western blot analysis

Western blot analysis was performed using anti-Oct-4 (C-20, Santa Cruz Biotechnology, Santa Cruz, CA, USA), anti-EGFP (Invitrogen Molecular Probes), anti-GST (B-14, Santa Cruz Biotechnology, Santa Cruz, CA, USA), anti-GAL4 (RK5C1, Santa Cruz Biotechnology), anti-Flag (M2, Sigma, St Louis, MO, USA), and anti-GAPDH (V-18, Santa Cruz Biotechnology) antibodies, and reactive bands were detected by chemiluminescence using Western Lightening (PerkinElmer Life Sciences, Boston, MA, USA).

### Establishment of stable cell lines and tumourigenic assays in nude mice

Details on the construction of ZHBTc4 ES cell line expressing EGFP have been previously reported (Lee *et al*, 2006). To generate stably expressing ES cell lines, pCAG-IP/EWS-Oct-4B-EGFP was linearised with *Pvu*I and 10 *μ*g of each was transfected into ZHBTc4 ES cells (4 × 10^6^) using the MicroPorator (Digital Bio, Seoul, Korea). At 48 h after electroporation, puromycin (Sigma) was added to a final concentration of 1 *μ*g ml^−1^ to select clones carrying stably integrated plasmid DNA. After selection of transfected ZHBTc4 cells, monoclonal cell lines were isolated by picking individual puromycin-resistant colonies. Tumourigenic assays were performed in nude mice as previously described (Kim *et al*, 1998a). In brief, 5-week-old athymic nude mice (CD1 nu/nu; Charles River, Wilmington, MA, USA) were pre-treated with tetracycline analogue doxycycline (10 *μ*g ml^−1^) in their drinking water for 2 weeks before injection. Then, 0.6 × 10^7^ cells from each clone in 100 *μ*l PBS were injected subcutaneously into nude mice and the mice continued to be exposed to doxycycline. Those mice that developed tumours were killed after 26 days.

## Results

### Structural features of EWS-Oct-4B

**Figure 1:**
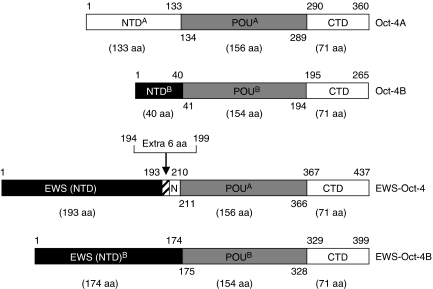
Structural comparison of EWS-Oct-4 alternative forms. Two different forms of the EWS-Oct-4 protein (EWS-Oct-4 and EWS-Oct-4B) and the human Oct-4 isoforms (Oct-4A and Oct-4B) are represented schematically. Amino acid (aa) positions are indicated above and below the schematic representing the proteins. In the case of EWS-Oct-4, the first 193 aa (residues 1–193) of EWS are fused to the truncated coding sequence (residues 123–360) of Oct-4A through an additional six aa sequence. This six aa sequence is encoded by the normal intron 6 of *EWS* in the fusion transcript that lacks the first 122 aa (residues 1–122) present in Oct-4A. In contrast, in the case of EWS-Oct-4B, the first 174 aa (residues 1–174) of EWS were fused to the POU DNA-binding domain (residues 136–360) of Oct-4A, in which neither the additional six aa sequence nor the N-terminal domain of Oct-4A is retained in EWS-Oct-4B. Functionally important domains of the Oct-4 isoforms and EWS-Oct-4 chimeras are indicated: NTD^A^, the N-terminal domain of human Oct-4A; POU^A^, POU DNA-binding domain (total 156 amino acids) of human Oct-4A; CTD, C-terminal domain of Oct-4; NTD^B^, N-terminal domain of human Oct-4B; POU^B^, POU DNA-binding domain (total 154 amino acids) of human Oct-4B; EWS (NTD), EWS N-terminal domain (residues 1–193) of EWS-Oct-4; Extra 6 aa, extra six amino acids; N, truncated N-terminal domain of Oct-4; EWS (NTD)^B^, EWS N-terminal domain (residues 1–174) of EWS-Oct-4B.

The *EWS-Oct-4* chimeric gene was originally isolated from bone and soft-tissue tumours (Yamaguchi *et al*, 2005). This chimeric gene encodes a protein in which the first 122 amino acids present in the NTD of Oct-4 are replaced by the N-terminal 193 amino acids of EWS through an additional six amino acid sequence encoded by the normal intron 6 of *EWS* (Figure 1). Recently, a different type of EWS-Oct-4 chimeric transcript, EWS-Oct-4B, was isolated in a hidradenoma of the skin and a mucoepidermoid carcinoma of the salivary gland (Moller *et al*, 2008). Sequence analysis of the *EWS-Oct-4B* gene revealed that a part of *EWS* exon 6 was fused in-frame to exon 2 of *Oct-4*, but unlike the EWS-Oct-4 fusion found in bone and soft-tissue tumours, neither the additional six amino acid sequence nor the NTD of Oct-4A was retained in the EWS-Oct-4B fusion (Figure 1). In addition, the POU domain of EWS-Oct-4B (POU^B^, total 154 amino acids) lacks two amino acids of the N-terminal sequence of the EWS-Oct-4 POU domain (POU^A^, total 156 amino acids). As a result of this particular chromosome translocation, the structure of the C-terminal portion of EWS-Oct-4B is more similar to the human Oct-4B isoform than to Oct-4A.

### EWS-Oct-4B is a nuclear protein

**Figure 2:**
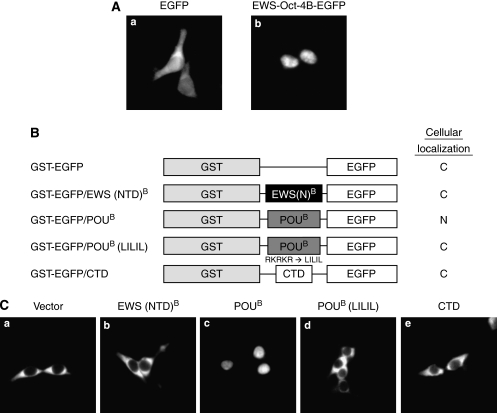
Mapping of the nuclear localisation signal of EWS-Oct-4B to the POU^B^ DNA-binding domain. (**A**) Subcellular localisation of EWS-Oct-4B. Cells (293T) grown on coverslips were transfected with mammalian expression vectors encoding (a) EGFP or (b) EWS-Oct-4B-EGFP. The subcellular localisation of the EWS-Oct-4B protein was determined by monitoring the location of the green fluorescence. (**B**) Schematic diagram showing EWS-Oct-4B truncation mutants of GST-EGFP fusion proteins. Subcellular localisation of the indicated truncation mutants was determined by monitoring the location of green fluorescence, and is indicated as N (nuclear localisation) or C (cytoplasmic localisation). (**C**) Subcellular distribution of EWS-Oct-4B deletion mutants. 293T cells were grown on coverslips under low-density conditions and transfected with expression plasmids for the indicated GST-EGFP-EWS-Oct-4B deletion mutants. The cells were fixed with an acetone/methanol mixture and EGFP was analysed by fluorescence microscopy.

We have shown previously that EWS-Oct-4 localises to the nucleus (Lee *et al*, 2007). As the structure of EWS-Oct-4B is more similar to human Oct-4B rather than to Oct-4A (Figure 1), and as Oct-4B is mainly localised to the cytoplasm (Lee *et al*, 2006), we decided to investigate the subcellular localisation of EWS-Oct-4B. As a first step, we transfected 293T cells with the pEGFP-N1 vector or with pEWS-Oct-4B-EGFP and carefully monitored the localisation of green fluorescence. In the transiently transfected cells, EGFP-tagged EWS-Oct-4B was clearly localised to the nucleus (Figure 2Ab), whereas EGFP alone was found in both the nucleus and cytoplasm (Figure 2Aa). These data indicate that EWS-Oct-4B is a nuclear protein, unlike human Oct-4B, which is mainly localised to the cytoplasm.

To map the region of EWS-Oct-4B responsible for its nuclear localisation, we generated a set of EWS-Oct-4B deletion mutants (Figure 2B). Because polypeptides with molecular masses of <40–50 kDa can passively diffuse into the nucleus (Breeuwer and Goldfarb, 1990), we fused the isolated functional domains of EWS-Oct-4B to GST and EGFP. The 293T cells were transfected with expression vectors for the GST-EGFP fusion proteins of the EWS-Oct-4B truncation mutants, and the localisation of EGFP was analysed by fluorescence microscopy. GST-EGFP/EWS (NTD)^B^ (Figure 2Cb) and CTD (Figure 2Ce) localised to the cytoplasm, whereas GST-EGFP/POU (Figure 2Cc) clearly localised to the nucleus. GST-EGFP was used as a control and localised to the cytoplasm of 293T cells (Figure 2Ca).

To define NLS further in the POU^B^ domain of EWS-Oct-4B, we generated a POU^B^ mutant in which several highly conserved basic amino acids, ^269^RKRKR^273^, were replaced with the ^269^LILIL^273^ sequence using site-directed mutagenesis. This region of basic amino acids was chosen as a putative NLS on the basis of earlier evidence that states that positively charged sequences are good candidates for nuclear targeting signals (Dingwall and Laskey, 1991). Consistent with our prediction, the substitution of ^269^RKRKR^273^ with the ^269^LILIL^273^ sequence resulted in the cytoplasmic accumulation of GST-EGFP/POU^B^ (Figure 2Cd). This result suggested that this cluster of basic amino acids in the POU^B^ domain does indeed function as an NLS of EWS-Oct-4B.

### EWS-Oct-4B is a less-potent transcriptional activator than EWS-Oct-4

**Figure 3:**
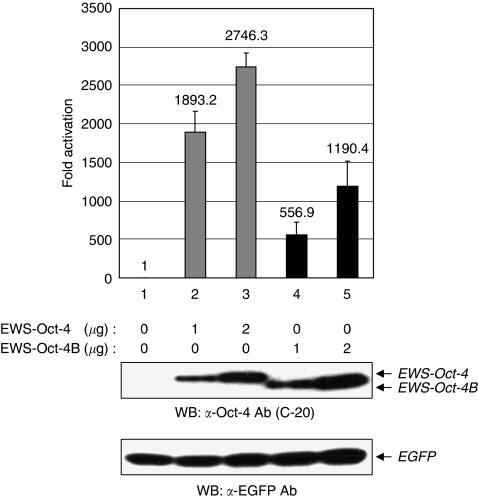
Comparison of transactivation potential by EWS-Oct-4B. Cells (293T) were cotransfected with expression vectors encoding the indicated amounts of EWS-Oct-4 (grey bars) or EWS-Oct-4B (black bars), the pOct-4(10 × ) TATA luc reporter plasmid, and the *Renilla* luciferase (top panel). Reporter activity was normalised with *Renilla* luciferase activity to correct for different transfection efficiencies. Fold induction is expressed relative to the empty expression vector. Each transfection was performed at least thrice independently and the mean values are plotted with their standard errors (±s.e., vertical bars). Extracts for luciferase assays were resolved using 12% SDS–PAGE, transferred to a PVDF membrane, and immunoblotted with anti-Oct-4 (C-20) (middle panel) or anti-EGFP (bottom panel) antibodies, as indicated.

To assess the transactivation potential of EWS-Oct-4B, we compared the transcription activities of EWS-Oct-4B and EWS-Oct-4 by cotransfecting their respective expression vectors with a reporter plasmid containing 10 copies of the Oct-4-binding site and a TATA box upstream of the luciferase gene (Lee *et al*, 2005). We also included a control plasmid consisting of a cytomegalovirus-driven *Renilla* luciferase gene. As shown in Figure 3, cotransfected EWS-Oct-4B caused a 1100-fold increase in reporter expression in 293T cells (Figure 3 top panel, bars 4 and 5) compared with an ∼2700-fold increase by EWS-Oct-4 (Figure 3 top panel, bars 2 and 3). Our results indicate that EWS-Oct-4B is a less-potent transcriptional activator than EWS-Oct-4. Western blotting of cell extracts from transfected cells showed that increasing amounts of EWS-Oct-4 and EWS-Oct-4B proteins were synthesised in response to increasing amounts of the corresponding plasmids (Figure 3 middle panel). In addition, western blot analysis showed that the difference in transactivation potential between EWS-Oct-4 and EWS-Oct-4B was not due to differences in the amounts of these proteins. The EGFP expression plasmid served as an internal control for monitoring transfection efficiency (Figure 3 bottom panel).

### EWS-Oct-4B similar to EWS-Oct-4 binds to an Oct-4 consensus sequence

**Figure 4:**
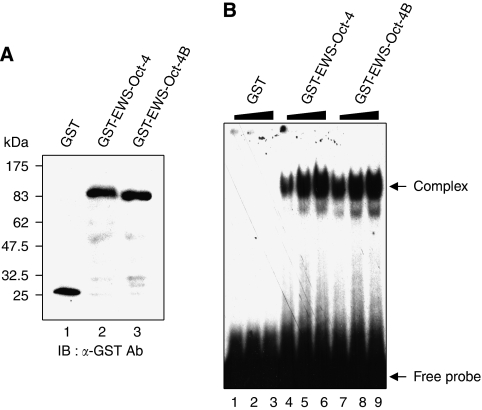
DNA-binding property of the EWS-Oct-4B chimera. (**A**) Immunoblot analysis of EWS-Oct-4B and EWS-Oct-4 to quantify GST fusion proteins. The GST-EWS-Oct-4 fusion proteins used in EMSAs were fractionated on 10% SDS–PAGE and visualised by western blotting with an anti-GST antibody (B-14, Santa Cruz Biotechnology). (**B**) EMSAs of the DNA-binding properties of EWS-Oct-4B and EWS-Oct-4. The EMSAs were performed using recombinant GST (lane 1, 0.15 *μ*g; lane 2, 0.45 *μ*g; lane 3, 1.35 *μ*g), GST-EWS-Oct-4 (lane 4, 0.15 *μ*g; lane 5, 0.45 *μ*g; lane 6; 1.35 *μ*g), or GST-EWS-Oct-4B (lane 7, 0.15 *μ*g; lane 8, 0.45 *μ*g; lane 9, 1.35 *μ*g), and radiolabeled probe, as described in Materials and Methods. The recombinant proteins used in each EMSA are indicated above the gel. Protein–DNA complexes were resolved on non-denaturing 4% polyacrylamide (acrylamide:bisacrylamide ratio, 37 : 1) gels run at 4°C in 0.5 × TBE (44.5 mM Tris-HCl, 44.5 mM boric acid, 1 mM EDTA). The positions of free probe and protein–DNA complexes are indicated.

The POU domain of Oct-4 is a conserved DNA-binding domain that binds as a monomer to the octamer sequence motif, 5′-ATGCAAAT-3′ (Botquin *et al*, 1998). Although there is considerable structural similarity between EWS-Oct-4B and EWS-Oct-4 (Figure 1), POU^B^, the DNA-binding domain of EWS-Oct-4B, lacks two amino acids of the N-terminal sequence found in the EWS-Oct-4 POU domain (POU^A^), and the structure of the EWS-Oct-4B C-terminal portion (POU plus CTD) is more similar to that of the Oct-4B isoform than to that of Oct-4A. Interestingly, the Oct-4B isoform does not bind to a probe carrying the Oct-4 consensus-binding sequence, and two separate regions of its NTD are responsible for inhibiting DNA binding (Lee *et al*, 2006). To determine whether EWS-Oct-4B binds the target sequence of Oct-4, we performed EMSAs. An oligonucleotide containing the consensus Oct-4 DNA-binding sequence (Botquin *et al*, 1998) was synthesised and used as target in the binding reactions. Glutathione-S-transferase fusions of EWS-Oct-4B and EWS-Oct-4 were expressed in *Escherichia coli*, purified, and coupled to glutathione–Sepharose beads. To quantify the amount of each protein used, affinity-purified GST, GST-EWS-Oct-4B, and GST-EWS-Oct-4 proteins were fractionated by SDS–PAGE, transferred to a PVDF membrane, and immunoblotted with an anti-GST antibody (B-14, Santa Cruz Biotechnology) (Figure 4A). Electrophoretic mobility shift assays were then performed by keeping the concentration of the Oct-4 probe constant and varying the amount of input protein. Protein–DNA complexes were formed with both EWS-Oct-4B and EWS-Oct-4 proteins (Figure 4B lanes 4–6 and 7–9), whereas GST alone hardly bound at all (Figure 4A lanes 1–3). Binding was specific, as these complexes were displaced by a 100-fold excess of cold oligonucleotide containing the Oct-4-binding site, but not by a 100-fold excess of cold mutant oligonucleotide containing a mutant Oct-4-binding sequence that is not recognised by Oct-4 (data not shown). These results indicate that, although EWS-Oct-4B shares its C-terminal portion with the human Oct-4B isoform, the DNA-binding specificity of EWS-Oct-4B resembles that previously defined for Oct-4A.

### EWS (NTD)^B^ is a less-potent transcriptional activation domain than EWS (NTD)

**Figure 5:**
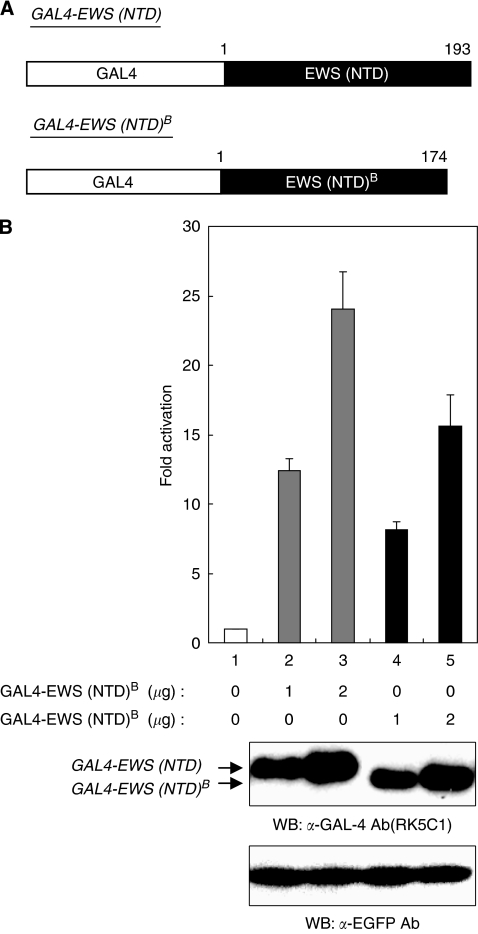
Transactivation potential of EWS (NTD)^B^. (**A**) Schematic representation of the GAL4-fusion expression plasmids used in this study. The expression vectors driving the production of GAL4-EWS (NTD) or GAL4-EWS (NTD)^B^ are shown. GAL4, GAL4 DNA-binding domain; EWS (NTD), EWS N-terminal domain of EWS-Oct-4; EWS (NTD)^B^, EWS N-terminal domain of EWS-Oct-4B. (**B**) Comparison of transactivation potential of EWS (NTD)^B^. The reporter plasmid, 5 × Gal4 TATA luc, was cotransfected with GAL4-EWS (NTD) or GAL4-EWS (NTD)^B^ into 293T cells (top panel). Luciferase activity was expressed as fold activation relative to the basal level observed with the reporter plasmid and the GAL4 DNA-binding domain alone (lane 1). Each transfection was performed independently at least thrice and the mean values are plotted with their standard errors (±s.e., vertical bars). Extracts for luciferase assays were resolved using 12% SDS–PAGE, transferred to a PVDF membrane, and immunoblotted with anti-GAL-4 (RK5C1) (middle panel) or anti-EGFP (bottom panel) antibodies as indicated.

Although protein–DNA complexes were formed with both EWS-Oct-4B and EWS-Oct-4 proteins (Figure 4) and both are localised to the nucleus (Figure 2), EWS-Oct-4B was a less-potent transcriptional activator than EWS-Oct-4 (Figure 3), suggesting that this difference could be due to the differences in their NTD s. To verify this hypothesis, we created fusion proteins in which the GAL4 DNA-binding domain was fused to the EWS (NTD) of EWS-Oct-4 or to the EWS (NTD)^B^ of EWS-Oct-4B (Figure 5A). The pG5 luc reporter contains five GAL4 DNA-binding sites upstream of the TATA box and was used as a reporter in these experiments. An expression vector pGAL4 (pM, Clontech Laboratories), which drives the synthesis of only the GAL4 DNA-binding domain, had no significant effect on the level of luciferase produced from pG5 luc when transfected into 293T cells (Figure 5B, lane 1). Interestingly, pGAL4-EWS (NTD) strongly activated luciferase production from pG5 luc by 25-fold (Figure 5B, lane 3), indicating that the EWS (NTD) of the EWS-Oct-4 protein has an intrinsic transcriptional activation property. On the other hand, the EWS (NTD)^B^ of EWS-Oct-4B (lane 5) activated luciferase production from pG5 luc by 15-fold when fused to the DNA-binding domain of GAL4. These results show that the EWS (NTD)^B^ of EWS-Oct-4B is capable of activating transcription, but is a less potent transactivator than the EWS (NTD) of EWS-Oct-4.

### Multiple domains are important for EWS-Oct-4B function

**Figure 6:**
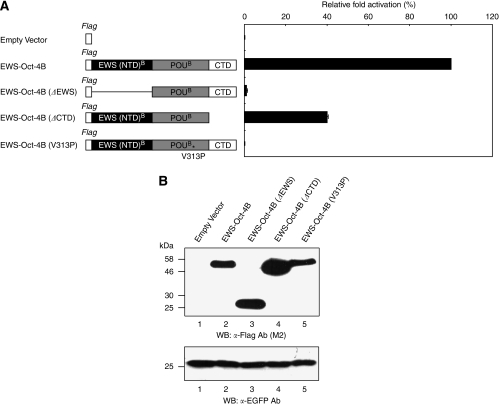
Functional regions of EWS-Oct-4B. (**A**) Transcriptional properties of EWS-Oct-4B deletion mutants. Shown on the left are schematic representations of EWS-Oct-4B deletion constructs. A reporter plasmid, pOct-4(10 × ) TATA luc, was cotransfected into 293T cells with various flag-tagged EWS-Oct-4B mutants. Relative transcriptional activation values are shown on the right as mean increases±s.e. relative to a value of 100% for transfection of EWS-Oct-4B. The results are the means of three independent experiments performed in duplicate. (**B**) Immunoblot analysis showing expression of the EWS-Oct-4B deletion mutants in transiently transfected cells. Total cell lysates were fractionated by 12% SDS–PAGE and visualised by western blotting with anti-Flag (M2, Sigma, top panel) or anti-EGFP (Invitrogen Molecular Probes, bottom panel) antibodies. The positions of pre-stained molecular weight markers (New England Biolabs, Hitchin, UK) are indicated to the left (kDa). Lane 1, empty vector; lane 2, EWS-Oct-4B; lane 3, EWS-Oct-4B (ΔEWS); lane 4, EWS-Oct-4 (ΔCTD); and lane 5, EWS-Oct-4B (V313P).

To define the critical regions within EWS-Oct-4B required for transactivation, we performed transient transfection experiments using flag-tagged EWS-Oct-4B deletions. The structures of the EWS-Oct-4B deletion mutants are shown schematically in Figure 6A. The data in the right panel show that deletion of the EWS (NTD)^B^ (named as EWS-Oct-4B (ΔEWS)) or a mutation of the POU DNA-binding domain (EWS-Oct-4B (V313P), which harbours a missense mutation in the POU DNA-binding domain converting ^313^Val to ^313^Pro) abolished transactivation activity, whereas removal of the CTD of EWS-Oct-4B (EWS-Oct-4B (ΔCTD)) reduced activity. We interpret these results to indicate that EWS (NTD)^B^ and CTD, as well as the DNA-binding activity of EWS-Oct-4B, are important for its full transactivation potential. The expression levels of all mutant proteins were examined by western blotting (Figure 6B, top panel). EGFP expression served as an internal control for monitoring transfection efficiency (Figure 6B, bottom panel).

### Regulation of endogenous Oct-4 downstream target genes by EWS-Oct-4B

**Figure 7:**
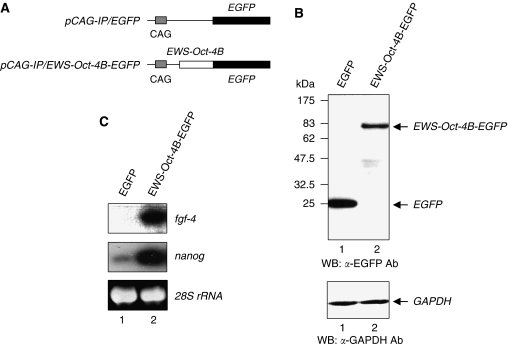
Transcriptional activation of Oct-4 downstream target genes by EWS-Oct-4B *in vivo*. (**A**) Schematic representation of the expression vectors. Expression vector pCAG-IP/EWS-Oct-4B-EGFP corresponds to EWS-Oct-4B fused to EGFP. The pCAG-IP/EGFP expression vector was used as a control. The CAG expression units (CAG) are indicated by shaded boxes, EWS-Oct-4B is represented by an open box, and EGFP is indicated by solid boxes. (**B**) Immunoblot analysis of EWS-Oct-4B expression in stably transfected ZHBTc4 ES cells. Total cell lysates (60 *μ*g protein) were fractionated by 12% SDS–PAGE and visualised by western blotting with anti-EGFP (Invitrogen Molecular Probes, top panel) or anti-GAPDH (V-18, Santa Cruz Biotechnology, lower panel) antibodies. (**C**) Induction of Oct-4 downstream target genes by EWS-Oct-4B *in vivo*. Northern blot analyses of *fgf-4* and *nanog* mRNAs were performed in ZHBTc4 ES cells expressing vector (lane 1) or EWS-Oct-4B fusion proteins (lane 2). Total RNA was fractionated on a 6% formaldehyde–1.5% agarose gel, transferred to a nylon membrane, and probed using mouse *fgf-4* (upper panel) or *nanog* (second panel) cDNAs, as described in Materials and Methods. Ethidium bromide (EtBr) staining of the agarose gel used for northern blotting shows that equal amounts of total RNA were loaded in each lane (lower panel). The stable cell lines from which the total RNAs used in the northern blot analysis were derived are shown above the panel.

To determine whether ectopic expression of EWS-Oct-4B could modulate the expression of endogenous Oct-4 downstream target genes such as fibroblast growth factor-4 (*fgf-4*) (Ambrosetti *et al*, 1997; Wang *et al*, 2003) and *nanog* (Kuroda *et al*, 2005; Rodda *et al*, 2005), we stably transfected Oct-4-null ZHBTc4 ES cells with pCAG-IP/EGFP and pCAG-IP/EWS-Oct-4B constructs (Figure 7A). We used the ZHBTc4 ES cell line because EWS-Oct-4B-positive human epithelial tumour cell lines or their equivalents are not available. In addition, it was convenient to introduce expression of the EWS-Oct-4B gene ectopically, because both the endogenous alleles of Oct-4 have been inactivated by gene targeting in the ZHBTc4 ES cell line, which also harbours the tetracycline-repressible mouse Oct-4 transgene (Niwa *et al*, 2000).

The amount of EWS-Oct-4B protein was determined by western blotting. The stable cell line carrying EWS-Oct-4B produced the EWS-Oct-4B protein as an EGFP fusion form (Figure 7B, lane 2). No EWS-Oct-4B-EGFP protein was detected in the control cell line (Figure 7B, lane 1). As shown in Figure 7C, expression of *fgf-4* and *nanog* was detected in tetracycline-treated ZHBTc4 ES cells expressing the EWS-Oct-4B chimeric protein (Figure 7C, lane 2), but not in treated cells expressing the EGFP vector alone (Figure 7C, lane 1). Thus, EWS-Oct-4B is capable of activating Oct-4 downstream target genes *in vivo*.

### ZHBTc4 ES cells harbouring EWS-Oct-4B have tumourigenic growth potential in nude mice

**Figure 8:**
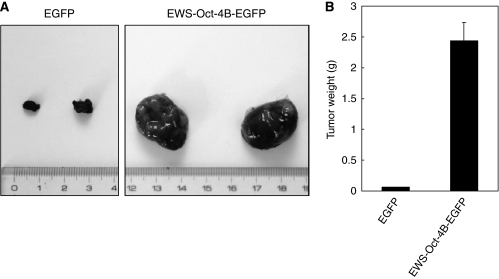
Characterisation of the effects of ZHBTc ES cells expressing EWS-Oct-4B in nude mice. (**A**) Effects of EWS-Oct-4B expression on the development of tumours in nude mice. Approximately 0.6 × 10^7^ Oct-4-null ZHBTc4 ES cells expressing EWS-Oct-4B-EGFP or EGFP, were suspended in 100 *μ*l PBS and injected into 5-week-old Balb/c athymic nude mice. Tumour development was observed and photographs were taken 26 days after injection. (**B**) Weight of tumours from nude mice injected with Oct-4-null ZHBTc ES cells expressing EWS-Oct-4B. Tumour weight in individual animals was measured at 26 days after injection (*n*=6) and is plotted as mean increases±s.e.

Finally, we examined the ability of the EWS-Oct-4B chimeric protein to induce tumours in nude mice. Nude mice were exposed to the tetracycline analogue, doxycycline, in their drinking water for 2 weeks before injection of EWS-Oct-4B cells, and the exposure was continued thereafter. As shown in Figure 8, all six mice injected with ZHBTc4 cells expressing EWS-Oct-4B developed large tumours within a relatively short latent period, showing that EWS-Oct-4B functions as an oncogene, at least as efficiently as EWS-Oct-4 (Lee *et al*, 2007) or Oct-4 (Gidekel *et al*, 2003). The same results were obtained with two other independent clones of ZHBTc4 cells expressing EWS-Oct-4B (data not shown). These results show that, similar to EWS-Oct-4 (Lee *et al*, 2007), overexpression of the EWS-Oct-4B chimeric protein is sufficient to induce tumourigenesis in nude mice.

## Discussion

Chromosome translocation is an important mechanism for creating oncogenes, and is the one that occurs frequently in human neoplasms (Rabbitts and Stocks, 2003). In this report, we have characterised the EWS-Oct-4B fusion protein, an alternative form of fusion between the *EWS* and *Oct-4* genes. This product is produced by chromosome translocation and is found in two types of human epithelial tumours (Moller *et al*, 2008). These tumours contain a characteristic t(6;22)(p21;q12) translocation, which results in the fusion of the amino terminal domain of EWS with the carboxy-terminal domain of human Oct-4. Here, we show that the alternative fusion protein, EWS-Oct-4B, is a nuclear protein that binds DNA with a sequence specificity indistinguishable from that of the parental Oct-4 or the original EWS-Oct-4 fusion product. In addition, this fusion gene is another powerful transforming gene, although it encodes a less-potent transcriptional activator than EWS-Oct-4.

The tumourigenic potential of the chimeric *EWS-Oct-4B* gene product is consistent with the idea that it has a crucial role in the formation of hidradenomas or mucoepidermoid carcinomas in humans. Although the EWS-Oct-4B form is revealed as a less-potent transcriptional activator than EWS-Oct-4 (Figure 3), the efficient and rapid growth of ZHBTc4 ES cells expressing EWS-Oct-4B protein in nude mice indicates that EWS-Oct-4B is also a potent oncogene (Figure 8). In human cancer, genes involved in controlling cell proliferation and survival are altered in ways that change either the behaviour or the amounts of proteins they produce. The chromosome rearrangement between *EWS* and *Oct-4* genes suggests the reactivation of silencing *Oct-4* gene expression as a form of *EWS-Oct-4B* fusion. Accumulating evidence shows that anomalous expression of *Oct-4* is involved in several human cancers. For example, the introduction of *Oct-4* into Swiss 3T3 cells causes their tumourigenic transformation and produces tumours in nude mice (Gidekel *et al*, 2003). Furthermore, several groups have reported that *Oct-4* is expressed in human tumours, including testicular germ cell tumours and breast carcinomas, and has a part in human cancer development (Jin *et al*, 1999; Monk and Holding, 2001; Gidekel *et al*, 2003; Looijenga *et al*, 2003). In addition, the activation of *Oct-4* in somatic tissues of adult mice, using a doxycycline-dependent expression system, results in dysplastic growth in epithelial tissues (Hochedlinger *et al*, 2005), indicating that abnormal expression of *Oct-4* may be the cause of tumourigenesis in somatic tissues.

Alternative splicing of human *Oct-4* mRNA gives rise to two different protein isoforms designated as Oct-4A and Oct-4B (Takeda *et al*, 1992). These isoforms are composed of 360 and 265 amino acids, respectively, of which the 225 amino acids at the carboxy-termini are identical. The CTDs (71 amino acids) of human Oct-4A and Oct-4B are identical, but the Oct-4B POU domain (total 154 amino acids) lacks two amino acids of the N-terminal sequence of the Oct-4A POU domain (total 156 amino acids). Outside these regions, Oct-4B has little similarity to Oct-4A. The C-terminal part of EWS-Oct-4B, isolated in a hidradenoma of the skin and a mucoepidermoid carcinoma of the salivary gland (Moller *et al*, 2008), was found to be more similar to that of Oct-4B than to that of Oct-4A. Similar to Oct-4B, the POU DNA-binding domain of EWS-Oct-4B lacks two amino acids of the N-terminal sequence of the EWS-Oct-4 POU domain. Interestingly, the human Oct-4B isoform does not bind to DNA carrying the typical Oct-4 consensus-binding sequence, is mainly localised to the cytoplasm, and does not activate transcription from an Oct-4-dependent promoter (Lee *et al*, 2006). However, unlike human Oct-4B, EWS-Oct-4B is a nuclear protein (Figure 2) that binds DNA with a sequence specificity indistinguishable from that of Oct-4A (Figure 4) and shows a potent transcriptional activation potential (Figures 3 and 7). Therefore, our data imply that the DNA-binding and transactivation properties of EWS-Oct-4B and Oct-4B differ, although there are structural similarities between these two proteins.

Although the DNA-binding specificity of EWS-Oct-4B resembles that previously defined for EWS-Oct-4 (Figure 4), we show that EWS-Oct-4B is a less-potent transactivator than EWS-Oct-4 (Figure 3). According to our previous report, the EWS NTD of EWS-Oct-4 contributes to its transcriptional activation function by providing a novel activation domain (Lee *et al*, 2007), which suggests that the difference in transactivation potential between EWS-Oct-4B and EWS-Oct-4 could be due to differences in their EWS NTDs. Consistent with this hypothesis, the EWS (NTD)^B^ of EWS-Oct-4B was capable of activating transcription, but was a less-potent transactivator than the EWS (NTD) of the EWS-Oct-4 fusion protein (Figure 5). The EWS NTD contains a large number of tyrosine, glutamine, alanine, serine, threonine, glycine, and proline residues, some of which are organised in a repeated and degenerate peptide motif with a frequently recurring serine–tyrosine dipeptide (NSYGQQS) that shares homology with the CTD of the large subunit of eukaryotic RNA polymerase II (Delattre *et al*, 1992). The amino acid sequence of the EWS (NTD) does not suggest any particular discrete region that might be important for transcriptional activation (Kim *et al*, 1998b); thus, it may be possible that the entire EWS (NTD) is necessary for its full transactivation potential.

It remains to be determined whether the structural differences between EWS-Oct-4B and EWS-Oct-4 are clinically significant in human cancer. In the case of Ewing's sarcoma, there are up to 18 possible types of in-frame *EWS-Fli-1* fusion transcripts and most of these isotypes have been observed *in vivo* (Zucman *et al*, 1993). Interestingly, regardless of tumour site, stage, or size, the survival rate of Ewing's sarcoma patients with type I EWS-Fli-1 fusion is markedly better than those in whom Ewing's sarcoma carries other types of EWS-Fli-1 fusions (Zoubek *et al*, 1996; de Alava *et al*, 1998; Lin *et al*, 1999). Although we have shown that EWS-Oct-4B regulates the expression of *fgf-4* and *nanog*, which are known potent mitogens (Figure 7), it remains to be seen whether the activation of *fgf-4* or *nanog* through EWS-Oct-4B is sufficient to produce the neoplastic phenotype found in the two types of epithelial tumours, hidradenoma of the skin and mucoepidermoid carcinoma of salivary glands. The functional characterisations of both EWS-Oct-4 and EWS-Oct-4B isoforms in human cancers should be of potential medical interest.

Recently, it was reported that abnormal expression of the EWS-Oct-4 chimera by chromosomal translocation induces an incomplete mesenchymal-to-embryonic transition (Makino *et al*, 2009). Because EWS-Oct-4 is a nuclear protein that binds DNA with a sequence specificity indistinguishable from that of the parental Oct-4 protein (Lee *et al*, 2007), and because ectopic expression of Oct-4 also causes dysplasia by inhibiting progenitor cell differentiation (Hochedlinger *et al*, 2005), it seems reasonable to speculate that the fusion of EWS NTD to the Oct-4 DNA-binding domain may produce a transforming chimeric product that inhibits cellular differentiation. Despite its reduced activity as a transcriptional activator, EWS-Oct-4B also regulated Oct-4 downstream target genes the promoters of which contain potential Oct-4-binding sites (Figure 7). Because the properties of EWS-Oct-4B are very similar to those of EWS-Oct-4, it would be interesting to investigate whether abnormal expression of EWS-Oct-4B in two types of epithelial tumours, hidradenoma of the skin and mucoepidermoid carcinoma of salivary glands, also contributes to the induction of the incomplete embryonic transition.

In conclusion, this study provides evidence that the EWS-Oct-4B fusion protein is an oncogene and that it is necessary for tumourigenesis. On the basis of our findings, we suggest that additional genes may cooperate with EWS-Oct-4 or be required for tumour progression. Although EWS-Oct-4B is a less-potent transactivator than EWS-Oct-4, it probably contributes to oncogenesis by activating key Oct-4 downstream target genes, such as *fgf-4* and *nanog*. It will be interesting to determine which downstream target gene(s) is/are critical for tumourigenesis, and whether EWS-Oct-4B collaborates with this/these gene(s) to generate human epithelial tumours.
